# Costs and cost-effectiveness of community health workers: evidence from a literature review

**DOI:** 10.1186/s12960-015-0070-y

**Published:** 2015-09-01

**Authors:** Kelsey Vaughan, Maryse C Kok, Sophie Witter, Marjolein Dieleman

**Affiliations:** Royal Tropical Institute (KIT), P.O. Box 95001, 1090 HA Amsterdam, The Netherlands; Queen Margaret University, Edinburgh, Scotland

**Keywords:** Community health workers, Cost-effectiveness, Costs, Literature review

## Abstract

**Objective:**

This study sought to synthesize and critically review evidence on costs and cost-effectiveness of community health worker (CHW) programmes in low- and middle-income countries (LMICs) to inform policy dialogue around their role in health systems.

**Methods:**

From a larger systematic review on effectiveness and factors influencing performance of close-to-community providers, complemented by a supplementary search in PubMed, we did an exploratory review of a subset of papers (32 published primary studies and 4 reviews from the period January 2003–July 2015) about the costs and cost-effectiveness of CHWs. Studies were assessed using a data extraction matrix including methodological approach and findings.

**Results:**

Existing evidence suggests that, compared with standard care, using CHWs in health programmes can be a cost-effective intervention in LMICs, particularly for tuberculosis, but also – although evidence is weaker – in other areas such as reproductive, maternal, newborn and child health (RMNCH) and malaria.

**Conclusion:**

Notwithstanding important caveats about the heterogeneity of the studies and their methodological limitations, findings reinforce the hypothesis that CHWs may represent, in some settings, a cost-effective approach for the delivery of essential health services. The less conclusive evidence about the cost-effectiveness of CHWs in other areas may reflect that these areas have been evaluated less (and less rigorously) than others, rather than an actual difference in cost-effectiveness in the various service delivery areas or interventions. Methodologically, areas for further development include how to properly assess costs from a societal perspective rather than just through the lens of the cost to government and accounting for non-tangible costs and non-health benefits commonly associated with CHWs.

## Introduction

In recent years, community health workers (CHWs) have received renewed attention in light of critical shortages in the health workforce and emphasis on strengthening primary healthcare systems for achieving global health goals [1–4]. CHWs are generally assumed to be a less expensive alternative compared with other cadres of health workers, notably with regard to salary and incentives as well as training costs. In parallel, more and more evidence has accumulated in recent years on the effectiveness of CHWs in delivery of essential health services in low- and middle-income countries (LMICs) [5–7]. However, studies assessing the costs and/or cost-effectiveness of CHW programmes are limited due both to data and methodological problems [1, 8, 9]. Therefore, we conducted an exploratory literature review to:

1. provide an overview of what is globally known about CHWs’ costs and cost-effectiveness
2. identify methodologies and elements of costs, effects and cost-effectiveness included in and excluded from studies to date
3. discuss appropriate methodologies for evaluating the costs and cost-effectiveness of CHWs.

As this was an exploratory review, no estimates in monetary terms (dollar values) are presented. Nonetheless, the overall conclusions about the costs and cost-effectiveness of CHWs will serve to inform policy dialogue around the role of CHWs in health systems, and findings about methodologies will encourage researchers to properly assess the costs and cost-effectiveness of such programmes.

## Methods

### Definitions

For the purpose of this literature review, the definition of CHW that we used is the following [6]: “Any health worker carrying out functions related to health care delivery; trained in some way in the context of the intervention, and having no formal professional or paraprofessional certificate or degree in tertiary education”. Costs are defined as the resources, either expended or foregone, associated with implementing a health programme or treatment. Cost-effectiveness as a study type is defined as “one form of economic evaluation where both the costs and consequences of health programmes or treatments are examined” [10]. When comparing two programmes or scenarios, intervention A is said to be more “cost-effective” than intervention B when programme cost per unit effectiveness for A is less than for B. “Cost-effective” may also refer to a comparison with a threshold or benchmark. In the rest of the paper, we refer to consequences as benefits or effectiveness, defined as the change in desired outcome due to the intervention or programme. We refer to final patient outcomes (change in health status and/or well-being) wherever possible; where not available, measurable intermediate patient outcomes (for example, number of patients visited and number of visits conducted) and measurable CHW provider outcomes (for example, improved CHW productivity) are used.

### Search strategy

**Figure 1:**
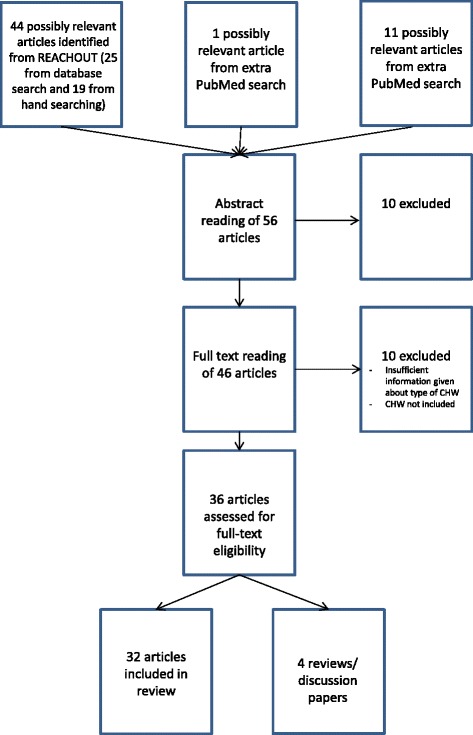
Flow chart of search strategy.

We used the search results from a larger, systematic review on factors influencing performance of close-to-community providers, which included searching the EMBASE, PubMED, Cochrane, CINAHL, POPLINE and NHS-EED databases for the period January 2003 to April 2013 [2] as well as a manual search of reference lists of all papers. This broader review included quantitative, qualitative and mixed method studies, all in English, about CHWs working in promotional, preventive or curative primary healthcare in LMICs. From that search, we extracted costing studies, studies that assessed the costs and effects of a single CHW intervention and economic evaluations assessing the costs and benefits of at least two CHW interventions. We conducted an additional search in PubMed for articles published during the same period to verify the existence of any further relevant papers (see Table 1); this search was later updated to include articles published from May 2013 to July 2015. The search strategy is summarized in Figure 1 while the full search strategy is presented elsewhere [2].

**Table 1:** **Search details of the supplementary search**

**PubMed**		**Results 16 July 2013 (for January 2003 to April 2013)**	**Results 1 August 2015 (for May 2013 to July 2015)**
#1	“community health worker” OR “community health workers” OR “community health workers”[MeSH]	1441	916
#2	“health economics” OR “economics, medical”[MeSH] OR “economic evaluation” OR “health care costs” OR “health resource allocation” OR “health resource utilization” OR costs OR “costs and cost analysis”[MeSH] OR “cost analysis” OR “cost-benefit analysis”[MeSH] OR “cost effectiveness” OR “cost effective” OR “health care costs” OR “cost benefit analysis” OR “cost-benefit analysis”[MeSH] OR costly OR costing OR price OR prices OR expenditure OR “health expenditures”[MeSH] OR “value for money” OR budget OR budgets OR DALYs OR QALYs OR “quality-adjusted life years”[MeSH]	96 561	64 724
#1 AND #2		134	113

### Review approach

Three reviewers jointly developed two separate data extraction matrices. The first matrix captured the study or model’s overall methodological approach as well as specifics regarding how costs, effects and cost-effectiveness of the CHW programme were assessed. The overall methodological approach included study design, perspective, time horizon, discounting, year of costing and currency, intervention and comparator(s), setting, scenarios, sensitivity analysis and software. In terms of costs, the data extraction form captured programme (training, recurrent, capital and overhead/indirect) and patient costs, costs averted, how costs are reported and data sources. A review of the outcomes included both patient and provider outcomes and was defined as final patient outcomes (change in health status and/or well-being), measurable intermediate outcomes (for example, number of patients visited and number of visits conducted) and measurable CHW provider outcomes (for example, improved CHW productivity). The cost-effectiveness measure was also indicated. Although elements of quality were included in our data extraction and analysis, our review did not exclude studies based on a full assessment of study quality, because of the high diversity in types and focuses of the studies.

The second matrix captured the study or model’s findings in terms of costs, outcomes and cost-effectiveness. Findings from any sensitivity analyses were also extracted. Systematic reviews were summarized in terms of main CHW-related findings.

**Figure 2:**
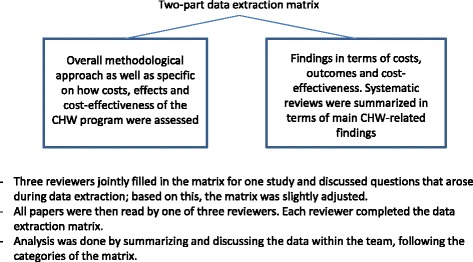
Review approach.

We piloted the abstraction process by having the three reviewers jointly analyse and discuss one article and then discuss as a team questions that arose during data extraction. All papers were then read and abstracted by a single reviewer. Each reviewer completed the data extraction matrix separately, and review results were compiled into a single matrix for analysis. Analysis was done by summarizing and discussing the data within the team, following the categories as presented above. For an overview of the review approach, see Figure 2.

## Results

### Search results

The search strategy resulted in 32 individual articles about 31 studies being fully reviewed using the data extraction matrix and four review/discussion papers summarized. Table 2 presents an overview of the methodological characteristics of included studies, while Table 3 outlines the included studies with their location, type of CHW, intervention description, perspective, cost components included, and any assessment regarding cost-effectiveness. These aspects of the included studies are discussed below.

**Table 2:** **Methodological characteristics of included studies**

**Methodological area**	**Details of included studies**
Study type	17 economic evaluations, often comparing CHWs with facility-based practice
	5 articles looked at the costs and benefits of a single intervention or programme
	10 articles included cost data only
Perspective	Provider or health system perspectives (*n* = 15)
	Wider societal perspectives (*n* = 14)
	Three studies did not specify the perspective taken
Time horizon	Only four studies included a time horizon greater than 1 year
	The others (*n* = 28) either did not specify a time horizon or used 1 year
Sensitivity analysis	17 studies performed a sensitivity analysis, the majority (*n* = 10) using a one-way or univariate analysis
	Variables used in the sensitivity analysis include the following: unit costs and quantities of provider and patient cost items, assumptions about training (varying the intensity, excluding one type of training and varying the cost of the training), varying discount and exchange rates, administrative support, useful life of capital items and effectiveness data, including CHW salaries, including inclusion of life years saved and deaths averted

**Table 3:** **Summary of key methodological points and cost and cost-effectiveness results of included studies**

**Study**	**Country**	**Type of CHW**	**Description**	**Type of study and perspective**	**Programme costs included**				**Patient costs included**	**Narrative conclusion on cost and/or cost-effectiveness**
					**Training**	**Capital**	**Recurrent**	**Joint/overhead**		
*Maternal health*										
Alem et al. 2012 [27]	Bangladesh	CHWs	Dissemination of health messages, identifying pregnancies, bringing pregnant women to birthing huts, accompanying them during their delivery and providing newborn care by CHWs.	Costing of CHW dropout from a provider perspective.	Yes	Yes	Yes	Yes	No	CHW dropout after training and working for 1 month leads to foregone health services as well as recruitment and training of replacements. With an additional investment double the initial investment per CHW, the organization reduces dropout, can make additional cost savings (not recruiting and training a replacement) and fewer services are foregone in the community.
Sutherland and Bishai. 2009 [39]	India	Village health workers (VHWs)	Simulation study on maternal health: prevention of PPH and anaemia by VHWs.	Cost-effectiveness study from a provider perspective.	Yes	No	Yes	No	No	Misoprostol prevention and treatment provided by VHWs are both more cost-effective than standard care (although standard care is not defined). Treatment is significantly more cost-effective than prevention in terms of cost per life saved.
Sutherland et al. 2010 [40]	India	VHWs	Simulation study on prevention of PPH by VHWs.	Cost-effectiveness study from a provider perspective.	Yes	No	Yes	No	No	Misoprostol prevention and treatment provided by VHWs are both more cost-effective than standard care (although standard care is not defined). Treatment is significantly more cost-effective than prevention in terms of cost per life saved.
Chin-Quee 2013 [26]	Zambia	CHWs	Family planning intervention by CHWs	Costs and benefits of a single intervention from a programme perspective.	Yes	No	Yes	No	No	Provision of injectable contraceptives by CHWs can be done at low cost when added to an existing community-based distribution package.
*Neonatal health*										
Borghi et al. 2005 [11]	Nepal	Women group facilitators	Maternal health intervention with women’s groups.	Economic evaluation with provider perspective alongside RCT	Yes	Yes	Yes	Yes	No	Women groups facilitated by lay health workers could provide a cost-effective way of reducing neonatal deaths compared to current practice.
Chola et al. 2011 [28]	Uganda	Peer supporters	Breastfeeding intervention delivered by local women trained as peer supporters.	Costing study from a local provider perspective	Yes	Yes	Yes	Yes	No	The use of local women trained as peer supporters to individually counsel women about exclusive breast feeding can be implemented in sub-Saharan Africa at a “sustainable cost”.
Sabin et al. 2012 [38]	Zambia	Traditional birth attendants (TBAs)	Neonatal healthcare delivered by trained TBAs.	Costing and cost-effectiveness study alongside RCT; financial analysis based on trial costs only then expanded to intervention economic costs from societal perspective	Yes	No	Yes	No	No	The strategy of using trained TBAs to perform the neonatal resuscitation protocol (NRP) and antibiotics with facilitated referral to a health centre (AFR) to reduce neonatal mortality was found to be highly cost-effective as compared to GDP per capita and per WHO guidelines in Zambia.
*Child health*										
Fiedler 2003 [16]	Honduras	Monitors/CHWs	Growth monitoring of children under two by CHWs. The CHW treats and refers children under five to health services.	Costing study from a health service perspective.	Yes	Unclear	Yes	No	No	CHW programme cost 11% of the facility-based alternative while saving outpatient visits and costs.
Fiedler et al. 2008 [17]	Honduras	Monitors/CHWs	Growth monitoring of children under two by CHWs. The CHW treats and refers children under five to health services.	Costing study from a health service perspective	Yes	Unclear	Yes	No	No	CHW programme cost 11% of the facility-based alternative while saving outpatient visits and costs.
Nonvignon et al. 2012 [19]	Ghana	CHWs	CHW home management of malaria using two different drugs, by voluntary community-based agents in Ghana.	Cost-effectiveness study with a societal perspective	Unclear	Yes	Yes	Unclear	Unclear	Home management of under-five fevers by trained, unpaid community volunteers through diagnosis and dispensing of antimalarials and/or antibiotics was found to be a cost-effective strategy (in terms of cost per DALY averted compared with threshold recommended by WHO) for reducing under-five mortality in this setting.
Prinja et al. 2013 [36]	India	Auxiliary nurse midwives (ANM), anganwadi workers (AWW) and accredited social health activists (ASHA)	Comparison of costs of integrated management of neonatal and childhood illnesses (IMNCI) and no IMNCI.	Economic evaluation from a programme perspective nested in an effectiveness trial	Yes	Yes	Yes	Yes	No	Implementation of IMNCI imposes additional costs to the health system; cost-effectiveness needs to be assessed in a comprehensive economic evaluation.
Puett et al. 2013 [37]	Bangladesh	CHWs	Comparison of home management of severe acute nutrition versus facility-based inpatient treatment.	Cos-effectiveness study from a societal perspective	Yes	Yes	Yes	Yes	Yes	Treatment of severe acute malnutrition by CHWs is highly cost-effective compared to facility-based treatment.
Tozan et al. 2010 [23]	Africa	CHWs	A community-based pre-referral artesunate treatment and referral programme by CHWs for children suspected to have severe malaria in areas with poor access to formal healthcare in rural Africa.	Cost and effects of single intervention from a provider perspective	Unclear	No	Yes	No	No	Pre-referral artesunate treatment delivered by CHWs is a cost-effective (as compared to GDP per capita and per WHO guidelines), life-saving intervention, which can substantially improve the management of severe childhood malaria in rural African settings.
*Tuberculosis*										
Clarke et al. 2006 [14]	South Africa	Lay health workers (LHWs)	Tuberculosis treatment adherence and counselling by trained LHWs on farms.	Cost-effectiveness analysis alongside RCT from a health district perspective	No	Unclear	Yes	No	No	Costs to public budgets can be substantially reduced while maintaining or improving case detection and treatment outcomes, by using farm-based LHWs.
Datiko and Lindtjørn 2010 [15]	Ethiopia	Health extension workers (HEWs)	HEWs administered DOT for 2 months during intensive phase at health post, gave out drugs on monthly basis during continuation phase.	Cost and cost-effectiveness as part of randomized trial from a societal perspective	No	Yes	Yes	Yes	Yes	Involving HEWs in TB treatment is cost-effective alternative to health facility delivery.
Floyd et al. 2003 [18]	Malawi	Guardians	Out-patient DOT at health facilities (by CHW) or by community member guardian (only new smear-negative patients), handing out drugs in an urban setting.	Cost and cost-effectiveness from a societal perspective	No	Yes	Yes	Yes	Yes	When new smear-positive and smear-negative patients were considered together, the new strategies were associated with a 50% reduction in total annual costs compared with the strategy used until end of October 1997 which did not require any direct observation of treatment.
Okello et al. 2003 [20]	Uganda	Community volunteers	DOT at community level by village-based volunteers.	Cost-effectiveness study from a societal perspective	Yes	No	Yes	Yes	Yes	Findings suggest there is a strong economic case for replacing hospital admission for the first 2 months of treatment followed by 6 months of daily unsupervised outpatient treatment with community-based care in Uganda, provided it is accompanied by strong investment in activities such as training, community mobilization and programme supervision.
Prado et al. 2011 [21]	Brazil	Trained guardians and CHWs	TB care in an urban setting.	Cost-effectiveness study from a societal perspective	Yes	Yes	Yes	Yes	Yes	Guardian-supervised DOT is an attractive option to complement CHW-supervised DOT.
Sinanovic et al. 2003 [22]	South Africa	CHWs/LHWs	New smear-positive pulmonary and retreatment patients receiving treatment for TB by CHWs/LHWs.	Economic evaluation from a societal perspective as part of a prospective cohort study	Yes	No	Yes	Yes	Yes	Community-based care is a cost-effective strategy for TB treatment compared with the facility alternative.
*Malaria*										
Chanda et al. 2011 [13]	Zambia	CHWs	CHWs using rapid diagnostic test for malaria in Zambia. Complicated malaria cases and non-malaria febrile cases were referred to the nearest health facility for further management. Uncomplicated malaria cases were treated by the CHW using artemisinin-based combination therapy (ACT).	Cost-effectiveness study from a provider perspective	No	Yes	Yes	Yes	No	Home management of uncomplicated malaria by CHWs was 36% more cost-effective than the standard of care at health facility level in this setting.
Conteh et al. 2010 [29]	Ghana	Community-based volunteers	Community-based volunteers delivered three different intermittent preventive treatments for malaria in children (IPTc) drug regimens to children aged 3–59 months.	Economic evaluation alongside RCT from a societal perspective	Yes	Yes	Yes	Unclear	Yes	Delivery of IPT for children by VVHWs is less costly than delivery by nurses working at outpatient departments or EPI outreach.
Hamainza et al. 2014 [24]	Zambia	CHWs	Home-based case detection and treatment of malaria with rapid diagnostic tests (RDTs) by CHWs versus facility care.	Costing study from a programme perspective alongside a longitudinal study.	Unclear	Unclear	Yes	Unclear	No	This way of delivering testing and treatment may be cost-effective at certain levels if community participation in regular testing is achieved.
Mbonye et al. 2008 [31]	Uganda	TBAs, drug-shop vendors, community reproductive health workers and adolescent peer mobilizers	Directly observed sulfadoxine-pyrimethamine (SP) therapy delivered by trained community resource persons to pregnant women through home visits during second and third trimester in a rural setting.	Cost-effectiveness study from both provider and patient perspectives	Yes	Yes	Yes	Yes	Yes	Community-based delivery of SP during pregnancy increased access and adherence to IPTp and was cost-effective according to World Bank criteria.
Onwujekwe et al. 2007 [41]	Nigeria	CHWs	Community members conducted treatment of presumptive malaria in uncomplicated adults and children.	Costs and benefits of a single intervention from both provider and community perspectives	Yes	Unclear	Yes	Unclear	Unclear	CHWs are an economically viable and “potentially cost-effective” (no comparator or benchmark given) source for providing timely, appropriate treatment of malaria in rural areas.
Patouillard et al. 2011 [33]	Ghana	VHWs	VHWS dispensed IPTc during three consecutive scheduled days from a central point of each village.	Costing study from a provider perspective alongside community randomized trial	Yes	Yes	Yes	Yes	No	Delivery of IPT for children by VHWs is less costly then delivery by nurses working at outpatient departments or EPI outreach.
*Other or multiple disease areas*										
Bowser et al. 2015 [34]	Mozambique	CHWs	Multi-year comparison of costs and benefits of delivery by CHWs of specialized targeted package of primary care interventions including family planning, maternal health, malaria, diarrhoea, pneumonia, TB, HIV, malnutrition and more.	Cost-effectiveness study taking a programme perspective	Yes	Yes	Yes	Yes	No	Using CHWs to deliver a range of primary care services can be less costly than other community-based programmes.
Buttorf et al. 2012 [12]	India	LHWs	LHWs/counsellors counselled on mental disorders.	Economic evaluation from a societal perspective alongside RCT	No	Yes	Yes	Unclear	Yes	LHW intervention resulted in cost savings from both a provider and patient perspective and achieved the same outcomes, making it more cost-effective than standard care at public primary care facilities.
Gaziano et al. 2014 [42]	South Africa	CHWs	This study compares CHWs visiting patients with uncontrolled hypertension two times a year with undefined usual care.	Cost-utility study using a Markov model, perspective undefined	Yes	Unclear	Yes	Unclear	No	The intervention is cost-saving, with the life cost being less than the annual cost due to reductions in non-fatal cardiovascular disease-related events.
Jafar et al. 2011 [30]	Pakistan	CHWs	CHWs provided advice at three monthly intervals on the importance of physical activity, diet and smoking cessation.	Cost-effectiveness study from a societal perspective alongside RCT	Yes	Yes	Yes	Yes	Yes	A combined intervention of HHE plus training of general practitioners to control high blood pressure is the most cost-effective solution as compared with other options.
Mahmud et al. 2010 [25]	Malawi	CHWs	CHWs using text messages delivered a variety of services including requesting medication deliveries, notifying patient deaths, sending appointment reminders, monitoring treatment adherence for TB DOTS and ART, queries and more.	Costing study with unspecified perspective (seems to be hospital)	No	Unclear	Yes	Unclear	No	m-health intervention delivered by CHWs resulted in both professional worker time and monetary savings compared with previous practice (a CHW programme without the m-health intervention).
McCord et al. 2013 [32]	Sub-Saharan Africa	CHWs	Various (diarrhoea, malaria, malnutrition, TB screening, pneumonia, management of pregnancy and health promotion).	Costing study from unspecified perspective (seems to be programme)	Yes	Yes	Yes	Yes	No	Comprehensive CHW subsystems can be deployed across sub-Saharan Africa at a cost that is modest compared with project costs of primary healthcare system.
Prinja et al. 2014 [35]	India	Auxiliary nurse midwives (ANMs), multi-purpose health workers (MPHWs) and accredited social health activist (ASHA) workers	Range of primary care services delivered by three types of CHWs at the sub-centre health facility level; study compares having one ANM with two ANMs.	Costing and cost-effectiveness study from a health system perspective	Unclear	Yes	Yes	No	No	Hiring a second ANM at the sub-centre level is very cost-effective given the incremental cost per unit increase in ANC coverage.

### Types of CHWs, setting and health priorities

In terms of types of CHWs included, the assessed articles included a range of CHW types and nomenclatures (see Table 3). Twelve out of 32 articles did not specifically discuss training or the duration was not specified; where mentioned, however, all CHWs received some type of training ranging from 1 day to 1 year [11–24].

The studies reported a variety of geographical areas and settings. Eighteen articles presented results from sub-Saharan Africa, nine from Asia and three from Latin America. Two articles included various countries in Africa (see Table 3). A number of different settings were included: home (*n* = 10), villages or general community (*n* = 7), health facility or health centre (*n* = 3) and workplace (*n* = 1). One study reviewed the experience of CHWs and mobile health (m-health). Several studies included CHWs operating in various settings (*n* = 4), while seven studies did not specify the exact setting.

Health priority areas addressed by CHWs included reproductive, maternal, newborn and child health (RMNCH, *n* = 13, including two reviews), tuberculosis (TB) (*n* = 6), malaria (*n* = 7) and a range of other disease areas or multiple areas including (problems regarding) hypertension, diarrhoea, malnutrition, pneumonia, common mental disorders and a range of primary care services (*n* = 7). CHWs working in RMNCH performed a wide variety of activities including basic curative activities, counselling and health promotion, referrals, prenatal care and support during home deliveries. CHWs involved in TB and malaria mainly administered directly observed therapy (DOT) of TB medicines and dispensed drugs. CHWs working in other disease areas were involved in different types of activities, ranging from health education and promotion, screening, diagnosis and management of some conditions to referrals.

### Costs

In terms of programme costs included, all but six studies [12, 13, 17, 22, 25, 26] clearly included the value of the CHWs’ time spent (either compensated or, for volunteers, opportunity cost or shadow price) and recurrent expenses such as materials, supplies, transport and supervision, although the individual unit quantities and costs were rarely reported. Eighteen studies included the value of capital items such as vehicles and equipment although the specifics were not always mentioned [11–13, 15, 18, 19, 21, 27–37]. Overhead costs were included in 17 studies [11, 13, 15, 18, 20–22, 27, 28, 30–37], for example, for TB, on the basis of the proportion of total health facility visits or inpatient days for which TB accounted. Three of the 13 studies used a flat rate of 15% or 30% [13, 30, 32].

Patient costs were included in 11 of the studies [12, 15, 18–22, 29–31, 37], including time for visits and hospitalization as well as transport, medicines, food and other expenses.

The studies relied on a wide range of data sources, including budget and expenditure files from health facilities, hospitals, districts, government price lists, patient questionnaires, literature, time sheets, payroll records, ministries of health and finance and project accounts.

Many of the studies did not estimate costs over a future time period; therefore, a discount rate was unnecessary. Four studies discounted costs at 3% [11, 19, 33, 38] and two others at 5% [27, 30]. The costing year ranged from 1996 to 2011, and all but one [23] reported in US dollars.

Studies reported costs in a number of different ways, including weighted mean costs [15], average programme costs [15], average costs [12, 13, 27, 39, 40], cost per activity [20, 21], cost per patient managed or treated [18, 22, 41], cost per child [16, 17, 23, 33, 35, 36], cost per inhabitant covered [32] or per capita [24] and total annual costs [19, 29, 31–34, 37]. Two studies estimated potential cost savings, from reduced facility visits [17] and reductions in non-fatal cardiovascular events [42], and another mentioned that the CHW intervention (TB care) may lead to a reduction in multi-drug-resistant TB and the related drug costs [14].

### Outcomes

Various outcome measures reported by included studies are presented in Table 4. As for costs, many of the studies did not estimate benefits over a future time period; therefore, a discount rate was unnecessary. Two studies mentioned discounting future benefits at 3% [19, 31] and another at 5% [30] in the base case or standard analysis. Data sources for outcomes included randomized trials, monitoring and evaluation systems, organizational and government offices, demographic surveillance systems and patient treatment registers. One study used assumptions about yearly incidence and disease progression [23].

**Table 4:** **Outcome measures**

Outcomes at the level of health status and well-being	
TB studies	Sputum smear results
	TB cure rate
	Treatment completion rate
	Treatment success rate
Malaria studies	Incidence of malaria and anaemia
MNCH studies	Neonatal mortality
	Deaths averted
	DALYs averted
	Incidence of acute PPH and severe PPH cases
	Anaemia cases averted
Other studies	Systolic blood pressure
	Presence/absence of depression or anxiety
Intermediate outcomes: patient level	
Number of patients registered or who received treatment	
Increased patient enrollment	
Number of patients counselled	
Number of patient visits made	
Number of referrals made	
Proportion of cases appropriately diagnosed and treated	
Number of doses taken by patients	
Weeks of exclusive breastfeeding	
Couple years of protection	
Intermediate outcome: health worker level	
Professional health worker time gained	

### Cost-effectiveness

Where assessed, the studies presented the cost-effectiveness of CHWs in terms of cost per visit [28], cost per patient or presumptive case successfully treated [14, 15, 20, 41], cost per patient cured [18, 21], cost per patient completing treatment [18, 37], cost per disability-adjusted life year (DALY) averted [23, 30, 37, 38, 40], cost per malaria case averted [29], cost per malaria case correctly diagnosed and treated [13], cost per case recovered [12], cost per couple-year of protection [26], cost per life year saved [11, 39] and cost per death averted [37]. Three studies reported the cost-effectiveness ratio comparing two interventions [19, 31, 35].

### Overall assessment of cost and cost-effectiveness by disease area

For ease of reporting, both costing and cost-effectiveness findings are presented below. For a summary of all results by disease area, see Table 3.

#### RMNCH

Findings about the costs and cost-effectiveness of CHWs for a number of different RMNCH conditions and for different types of activities are generally positive. For maternal health, misoprostol prevention and treatment provided by village health workers were found to be more cost-effective than standard care (although standard care was not defined). Treatment was also found to be significantly more cost-effective than prevention in a simulation setting (looking at cost per life saved only) [39, 40]. With regard to family planning, Chin-Quee et al. found the cost of adding an intervention to deliver injectable contraceptives to an existing community-based distribution package to be “low” [26]. In the area of neonatal health, women groups facilitated by lay health workers (LHWs) and trained traditional birth attendants (TBAs) were found to be cost-effective ways of reducing neonatal deaths compared to current practice [11, 38]. The use of local women trained as peer supporters to individually counsel women about exclusive breast feeding was found to be implementable in sub-Saharan Africa at a “sustainable cost” [28].

For child health, CHWs were found to be cost-effective for reducing under-five mortality and resulted in cost savings compared to the facility-based alternative for under-five child growth monitoring, counselling, curative care treatment and free-of-charge medicines as well as home visits as needed [16, 17, 19]. A study from India found implementation of integrated management of neonatal and child illnesses by CHWs imposed additional costs to the health system, but could not draw a conclusion about cost-effectiveness [36], while a study from Bangladesh on community management of severe acute malnutrition found the practice to be more cost-effective than facility-based inpatient treatment [37]. Two reviews of literature on the use of lay and community health workers in vaccination programmes by Corluka et al. [43] and Pegurri et al. [44] found these workers to be more cost-effective options than the comparator which did not include LHWs, including in an outreach setting.

#### TB

Studies from Brazil [21], Ethiopia [15], Malawi [18], South Africa [14, 22] and Uganda [20] found that using CHWs during the non-hospitalized phase of TB treatment is a cost-effective alternative to facility-based treatment. CHWs were found to reduce the cost per patient successfully treated and cured anywhere from 40% to 74% compared with facility-based provision. Okello et al. point out the importance of proper training and supervision in achieving success [20].

#### Malaria

Results are limited but generally positive from studies in favour of the cost-effective use of CHWs for malaria programmes compared with regular care. Studies found the delivery of intermittent preventive treatment (IPT) of malaria for children by village health workers was less costly then delivery by nurses in outpatient departments or immunization outreach [29, 33]; community-based delivery of sulfadoxine-pyrimethamine (SP) during pregnancy increased access, improved adherence to IPT and was cost-effective according to World Bank criteria [31]; and home management of uncomplicated malaria by CHWs was 36% more cost-effective than the standard care in health facilities [13]. The use of pre-referral artesunate for the treatment of childhood malaria by CHWs was found to be a cost-effective (according to WHO guidelines comparing cost per DALY averted with gross domestic product (GDP) per capita), life-saving intervention with potential application in rural African settings where CHW programmes are already in place (comparing cost per DALY averted with GDP per capita, according to WHO guidelines) [23]. A study from Zambia looking at active and passive case detection by CHWs including testing and treatment concluded that the programme may be cost-effective when community participation in regular testing reached certain levels [24]. Additionally, based on results from two villages, Onwujekwe et al. concluded that starting up a CHW programme for malaria control nationwide in Nigeria is potentially “cost-effective”, although no comparator or benchmark was given [41].

#### Other health priority areas

For wider primary care, studies have found that CHWs increased the coverage and equity of service delivery at low cost compared with alternatives, that using CHWs can be less costly than other community-based programmes and that comprehensive CHW subsystems can be deployed across sub-Saharan Africa at a modest cost compared with the project costs of a primary healthcare system [9, 32, 34]. A study from India further found that adding an additional primary care community-based health worker to the lowest level of the health facility was cost-effective, though results were only be measured in terms of cost of increasing ANC coverage [35].

Findings related to the cost-effectiveness of CHWs for other disease areas were limited but generally favourable to the use of CHWs to control hypertension (although in one study best results were achieved when combined with general practitioner training as well) [30, 42] and for interpersonal therapy and case management of patients with mental disorders [12]. Additionally, an m-health intervention implemented by CHWs on a variety of healthcare topics including treatment adherence monitoring, appointment reminders and emergency care resulted in monetary as well as time savings [25].

Overall, the results of this analysis are in line with findings from the review/discussion papers included in this study. Perry et al. found that where the cost-effectiveness of CHW-provided interventions is compared with that of facility-based interventions, the CHW-provided interventions are generally found to be more cost-effective [45], and Walker et al. found CHWs working in primary healthcare, vaccination and TB control programmes increase the coverage and equity of service delivery at low cost compared with alternative modes of service organization [9].

## Discussion

This review has found promising evidence in favour of the cost-effectiveness of CHWs as compared with standard practice or alternative delivery models or when comparing cost-effectiveness findings with a benchmark such as GDP per capita, although results should be interpreted with the understanding of both minor and major methodological challenges.

### Methodological issues and limitations

This analysis of 36 articles and reviews from the period January 2003 to July 2015 has revealed the variety of methodological approaches used to assess the costs and cost-effectiveness of CHWs, which limits both comparability and generalizability. Additionally, many articles did not provide sufficient details about study design or methodological assumptions, such as time horizon and study perspective, data quality and sources, limiting their usefulness. However, these were often earlier articles and may reflect the newness of economic evaluation methods. Many studies also failed to recognize the limitations of their data or question the quality. A mixed methods approach to costing and cost-effectiveness studies could enhance insight on the functioning and community-perceived value of CHWs and therefore add much-needed depth to a costing or cost-effectiveness study.

The issue of perspective has emerged as an important methodological challenge in this review: approximately half the studies in this review took a provider or health service perspective. Because of the nature of CHWs, taken from and embedded in the community, as well as health economics methodologies developed to date, current ways of assessing costs and benefits of these programmes (including use of the provider or government perspective) fail to capture many of the important societal costs and benefits associated with CHWs, such as social capital and trust as identified by Walker et al. [9] and improved relationships between patients and care providers. These aspects may have fallen outside the purview of economic evaluations to date because they are not monetizable, but leaving them out means we are failing to capture the true costs and benefits of CHWs in costing studies and economic evaluations. On the issue of perspective, in some cases, CHWs might reduce patient costs (for example, for TB, where patients receiving treatment in the community no longer have to travel to health facilities), making it important to take a wider perspective. And while our review did not touch on financial versus economic costs (how much the project or programme actually pays compared with the overall cost of the project or programme), this is a closely related issue. Additionally, as CHWs often operate as part of larger healthcare teams, it would be desirable to assess their cost-effectiveness as part of the broader health system in which they operate rather than as stand-alone programmes.

Finally, given the large number of CHW programmes, many operating already for decades, this review also reveals that the cost and cost-effectiveness of many CHW programmes have not been extensively and systematically assessed. As CHWs grow in popularity and are incorporated in human resources for health policies and plans in different countries, the need for well-designed and conducted costing and cost-effectiveness studies becomes particularly important.

### Cost and cost-effectiveness issues

This review has found evidence supporting the cost-effective use of CHWs, particularly in the area of TB; there are also studies supporting the cost-effective use of CHWs in the areas of RMNCH, malaria and other disease areas, although their methodology and quality of evidence are less strong. However, even where there is evidence suggesting a better cost-effectiveness of CHWs compared to other service delivery models, results should be interpreted with caution. The reviewed studies used very different methodologies; they compared CHWs to different cadres of health workers, and sometimes, there was no comparator. Furthermore, the studies in this review included and excluded different costs: for example, often they did not include the important and sizeable training and supervision or recruitment and retention costs related to CHWs. Additionally, volunteer time was valued differently in different studies and sometimes excluded altogether. Effectiveness of CHWs was also measured differently in different studies.

On the issue of comparability and generalizability, one fundamental challenge with comparing or generalizing CHW costing and cost-effectiveness findings is the varying nature of CHWs themselves. Although often lumped together, there is a wide typology of CHW models worldwide, with training and competencies varying enormously. Studies should include more details about the type of CHW being assessed and their context, and these differences should be taken into consideration when attempting to compare results [46].

The majority of articles reviewed documented CHW involvement in short-term or limited duration TB, malaria and RMNCH programmes, reflecting the use of CHWs in some countries for specific health areas or conditions. However, evidence is more limited about the costs or cost-effectiveness of CHWs who take on responsibilities across a wider range of disease areas or conditions and on the long-term cost-effectiveness and systemic implications of these programmes. A study by Alam et al. on maternal health from Bangladesh found retention of CHWs to be a problem, and the cost associated with dropout was significant, leading the programme to be less sustainable [27]. Cost-effectiveness analyses are often presented as snapshots of a certain short period of time, while longer term issues of retention and sustainability should be considered as well.

Further mixed method research is needed to better understand why CHWs are sometimes cost-effective and sometimes not and if there are fundamental aspects of different health areas that lend themselves to a cost-effective use of CHWs. For example, it could by hypothesized that CHWs are cost-effective in the area of TB because the activities performed are limited and easy to standardize, whereas CHW activities in the area of RMNCH may be more varied. It would be interesting to examine CHW cost-effectiveness on the different components of RMNCH separately (for example, antenatal care and deliveries separately) and analyse cost and cost-effectiveness differences between these activities. Additionally, research is needed to understand the impact of the task sharing on efficiency, costs and cost-effectiveness of both the programme from which the tasks were split, the CHW and the system as a whole [47].

Besides the limitations of the reviewed studies, limitations of this review itself should be taken into consideration. Publication bias is a potential issue; some relevant studies may have been missed if they were not identified by the larger search from which these results were taken or the supplementary PubMed search, and we did not consider grey literature. We have also not specifically evaluated the quality of the reviewed studies, though the review points out methodological shortcomings of the reviewed studies as a whole.

## Conclusions and policy implications

This literature review suggests that using CHWs in health programmes can be a cost-effective intervention in some settings, particularly for TB, with less strong evidence but promising indications of cost-effectiveness in RMNCH and malaria. These findings may relate to the fact that some areas have been evaluated less (and less rigorously) than others, rather than reflecting an actual difference in cost-effectiveness in the various service delivery areas or interventions.

Notwithstanding the caveats mentioned above about the heterogeneity of the studies and methodological weaknesses, this review shows that CHWs programmes have potential to represent good value for money for governments and donors for delivery of essential health services in LMIC. In developing or scaling up CHW programmes, however, more attention needs to be given to understanding costs and cost-effectiveness from both a government and societal perspective and to integrating community health workers in national healthcare systems in terms of employment, supervision, support and career development [48, 49].

## Abbreviations

CHW: Community health worker
DALY: Disability-adjusted life year
GDP: Gross domestic product
LHW: Lay health worker
LMIC: Low- and middle-income country
RMNCH: Reproductive maternal, newborn and child health
TB: Tuberculosis
TBA: Traditional birth attendant
